# Serum 25(OH)D3 and advanced liver fibrosis in type 2 diabetes: a Chinese hospital cohort with NHANES validation and mortality follow-up

**DOI:** 10.3389/fnut.2026.1820737

**Published:** 2026-07-22

**Authors:** Guohong Zhao, Jiaoqian Yang, Li Zhang, Shuo Yang, Duolin Niu, Meitong Yang, Zhaowei Gao, Fenghui Ma, Bin Gao

**Affiliations:** 1 Department of Endocrinology, Tangdu Hospital, The Fourth Military Medical University, Xi'an, China; 2 Department of Clinical Laboratory, Tangdu Hospital, The Fourth Military Medical University, Xi'an, China; 3 Department of Health Management, Tangdu Hospital, The Fourth Military Medical University, Xi'an, China

**Keywords:** 25-hydroxyvitamin D3, liver fibrosis, mortality, NAFLD, type 2 diabetes mellitus

## Abstract

**Background:**

Liver fibrosis is an important predictor of mortality in patients with type 2 diabetes mellitus (T2DM). Current clinical practice relies on total 25-hydroxyvitamin D, which does not distinguish between its two main circulating isoforms, 25(OH)D2 and 25(OH)D3, although these metabolites may differ in their associations with liver fibrosis and long-term prognosis.

**Methods:**

We analyzed two independent T2DM cohorts: hospitalized Chinese patients with T2DM (*n* = 1,978) and US adults with T2DM from the National Health and Nutrition Examination Survey (NHANES) 2007–2018 (*n* = 4,616). Serum 25(OH)D2 and 25(OH)D3 were quantified by liquid chromatography–tandem mass spectrometry (LC–MS/MS). Isoform-specific associations with advanced fibrosis, defined by the NAFLD Fibrosis Score (NFS) and Fibrosis-4 index (FIB-4), were examined using multivariable logistic regression and restricted cubic spline (RCS) analyses. In NHANES, multivariable Cox proportional-hazards models were used to evaluate all-cause, cardiovascular, and diabetes-related mortality according to vitamin D status.

**Results:**

In both cohorts, lower serum 25(OH)D3 was non-linearly associated with a higher risk of advanced liver fibrosis. By contrast, 25(OH)D2 showed no protective association; in NHANES, the highest tertile was associated with a higher risk of NFS-defined fibrosis (OR = 1.41, *P* = 0.003). RCS analyses suggested cohort-specific apparent inflection regions for 25(OH)D3 at 11.88 ng/mL in the Chinese cohort and 23.86 ng/mL in NHANES; these exploratory estimates were not intended as clinical cut-offs. In longitudinal NHANES analyses, lower 25(OH)D3 was associated with higher cause-specific mortality among participants with advanced fibrosis; however, estimates within the FIB-4 high-risk stratum were based on sparse events and were therefore regarded as exploratory and interpreted with caution. Bioinformatic analysis implicated the AGE–RAGE and PI3K–Akt signaling pathways as candidate mechanisms.

**Conclusion:**

Lower serum 25(OH)D3, but not 25(OH)D2, was associated with advanced liver fibrosis in T2DM after multivariable adjustment. Among participants with advanced fibrosis, lower 25(OH)D3 was also associated with higher cardiovascular mortality, although cause-specific estimates based on sparse events were exploratory. These findings may support further investigation of isoform-specific 25(OH)D3 assessment for risk stratification in this population.

## Graphical Abstract

**Figure:**
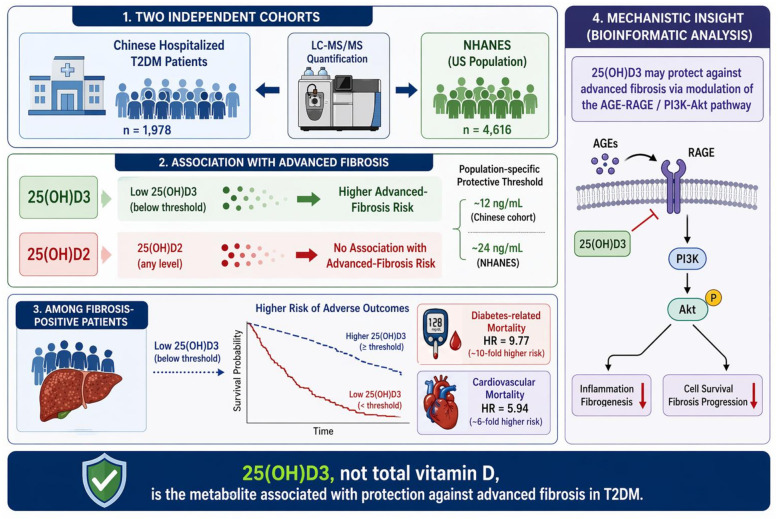

## 1 Introduction

Metabolic dysfunction-associated steatotic liver disease (MASLD), formerly referred to as nonalcoholic fatty liver disease (NAFLD), has emerged as a major chronic liver condition linked to metabolic dysfunction. Because the fibrosis scores and external evidence used in this study were developed and validated under the NAFLD framework, we use the term NAFLD throughout the present analysis for consistency. The disease spectrum ranges from simple steatosis to steatohepatitis, potentially progressing to irreversible fibrosis, cirrhosis, and hepatocellular carcinoma (HCC) (1). Based on recent global meta-analytic estimates, NAFLD affects approximately 30% of adults worldwide (2). This burden is particularly amplified in patients with T2DM, among whom a recent NAFLD meta-analysis estimated the pooled prevalence to be approximately 65% (3). Because T2DM is closely linked to liver injury progression, identifying modifiable risk factors to slow disease progression in this high-risk phenotype is a clinically important objective (3, 4).

Liver fibrosis serves as an important determinant of long-term prognosis in the natural history of NAFLD. As a subset of patients progresses to steatohepatitis, the risk of adverse hepatic outcomes escalates sharply (1, 5, 6). Consequently, the early identification and dynamic monitoring of advanced fibrosis are central challenges in clinical management. While liver biopsy remains the reference standard, its invasive nature and cost limit its routine use. Thus, non-invasive scoring systems such as the NAFLD Fibrosis Score (NFS) and Fibrosis-4 index (FIB-4) have been widely adopted. Scores exceeding validated high-risk thresholds (e.g., NFS > 0.676 and FIB-4 > 3.25) effectively identify patients with a high fibrotic burden and correlate with adverse outcomes and mortality (7, 8).

Beyond its canonical role in calcium and bone homeostasis, vitamin D exerts pleiotropic biological effects—including immune modulation, anti-inflammatory and antioxidant activity, endothelial regulation, and the control of cellular proliferation and differentiation—that are recognized across multiple organ systems (9–11). These extra-skeletal actions are particularly relevant to the liver, where the vitamin D receptor is expressed on hepatocytes, Kupffer cells and hepatic stellate cells, and has been implicated in the regulation of hepatic inflammation, insulin sensitivity and fibrogenesis. An overview of vitamin D metabolism, from cutaneous/dietary precursors through the two circulating 25(OH)D isoforms quantified in this study to downstream VDR- and RORα/γ-mediated signaling, is provided in Supplementary Figure S1.

Vitamin D deficiency has been implicated in the pathogenesis of NAFLD, potentially modulating liver injury through anti-inflammatory and immunoregulatory mechanisms (1, 12). However, a critical precision gap remains in the translational literature: the failure to distinguish between vitamin D isoforms. While ergocalciferol (25(OH)D2) and cholecalciferol (25(OH)D3) possess distinct bioavailability and physiological potency, current clinical paradigms predominantly rely on “total vitamin D” levels (13, 14). This generalized approach likely obscures the specific association of 25(OH)D3 with lower fibrosis risk and may also mask the absence of a comparable protective association for 25(OH)D2. The precise resolution of these metabolites requires liquid chromatography–tandem mass spectrometry (LC–MS/MS), yet large-scale studies using this reference analytical method to dissect isoform-specific effects on liver fibrosis remain limited (15).

To address this unmet need for precision management, we conducted a multi-population analysis. We analyzed clinical data from Chinese patients with T2DM quantified by LC–MS/MS and sought external validation in the NHANES database. Our objective was to evaluate the associations of distinct vitamin D metabolites with fibrosis scores (NFS and FIB-4), and to examine whether vitamin D status modified the relationship between advanced fibrosis and long-term mortality. The findings may inform future studies of isoform-specific vitamin D assessment in this high-risk population.

## 2 Materials and methods

### 2.1 Study design and participants

This study employed a multi-population observational design using two independent data sources: an internal hospital dataset for primary cross-sectional analysis and the National Health and Nutrition Examination Survey (NHANES, 2007–2018 cycles) for external validation and longitudinal survival analysis. The internal cohort initially comprised 3,375 patients diagnosed with T2DM according to World Health Organization (WHO) criteria, aged ≥18 years, and attended Tangdu Hospital, The Fourth Military Medical University (also known as the Second Affiliated Hospital of Air Force Medical University) between April 1, 2023, and June 30, 2025. Exclusion criteria were rigorously applied to both cohorts to ensure data quality and minimize confounding, specifically ruling out patients with positive hepatitis B/C serology, established cirrhosis, decompensated liver disease, other severe malignancies, or current use of medications affecting vitamin D metabolism. Ultimately, 1,978 eligible participants from the internal hospital cohort were included in the final analysis. The NHANES external cohort consisted of non-institutionalized U.S. adults aged ≥20 years with T2DM from the 2007–2018 cycles, with mortality tracked via the National Death Index (NDI). Participants were excluded if they were pregnant, had cancer, lacked serum vitamin D metabolite data, NFS, FIB-4, or mortality information, or did not meet the T2DM definition. The final NHANES analysis included 4,616 adults with T2DM. Patient selection flowcharts for the hospital and NHANES populations are presented in Supplementary Figures S2, S3, respectively.

### 2.2 Measurement of serum vitamin D metabolites

Serum concentrations of total 25-hydroxyvitamin D [25(OH)D] and its major isoforms were quantified using liquid chromatography–tandem mass spectrometry (LC–MS/MS), which is widely regarded as a reference method for vitamin D metabolite quantification. In the internal hospital cohort, serum 25(OH)D2 and 25(OH)D3 were measured using an LC–MS/MS platform (Shimadzu LCMS-8040CL) to ensure isoform-specific detection. In NHANES, vitamin D metabolites were also measured by LC–MS/MS according to the official laboratory protocol, including quantification of 25(OH)D3, 25(OH)D2, and epi-25-hydroxyvitamin D3 [epi-25(OH)D3] (16). Total 25(OH)D was defined as the sum of 25(OH)D3 and 25(OH)D2, excluding the C3-epimer. Because the internal hospital cohort and NHANES were analyzed in different laboratory settings, the analytical platforms and calibration procedures were not assumed to be identical. The laboratory methods for NHANES were based on publicly available NHANES documentation (16).

### 2.3 Assessment of liver fibrosis and mortality outcomes

The primary exposure variables were the non-invasive liver fibrosis scores NFS and FIB-4. These were calculated using standard clinical parameters:

- NFS = −1.675 + 0.037 × Age (years) + 0.094 × BMI + 1.13 × Impaired Fasting Glucose/Diabetes (Yes = 1, No = 0) + 0.99 × AST/ALT – 0.013 × Platelet Count (×10^9^/L) – 0.66 × Albumin (g/dL). For consistency with the original NFS formula, the variable “impaired fasting glucose/diabetes” was coded according to the original definition; in both T2DM cohorts, this term was uniformly coded as present.

FIB-4 = [Age (years) × AST (U/L)] / [Platelet Count (×10^9^/L) × √ALT (U/L)].

For the NHANES cohort, primary outcomes included all-cause, cardiovascular disease (CVD), and diabetes-related mortality. Mortality status was ascertained by linking NHANES data to the NDI through December 31, 2019. Diabetes-related mortality was defined based on ICD-10 codes E10–E14 in the linked mortality file, and cardiovascular mortality was defined using ICD-10 codes I00–I09, I11, I13, and I20–I51.

### 2.4 Covariates

To control for confounding, we adjusted for sociodemographic factors (age, sex, marital status), lifestyle indicators (alcohol, smoking), anthropometrics (BMI), and clinical markers [HbA1c, TG, LDL-C, hypertension, coronary heart disease (CHD)]. For the internal cohort, seasonal variation in vitamin D was accounted for by categorizing the examination date (Spring to Winter). A detailed list of covariates for both cohorts is provided in Supplementary Table S1.

### 2.5 Statistical analysis

Descriptive statistics and univariate analysis: Continuous variables were assessed for normality using the Shapiro–Wilk test. Variables with approximately normal distributions are reported as mean ± standard deviation (SD); skewed variables are reported as median (interquartile range, IQR). Categorical variables are presented as *n* (%). Between-group comparisons for continuous variables (NFS ≤ 0.676 vs. > 0.676; FIB-4 ≤ 3.25 vs. > 3.25) were performed using the independent-samples *t*-test or the Mann–Whitney U test as appropriate. Categorical variables were compared using Pearson's chi-square test or Fisher's exact test when indicated, and all tests were two-sided with *P* < 0.05 considered statistically significant.

Multivariable regression and non-linear analysis: The primary association between serum vitamin D metabolites (total 25(OH)D, 25(OH)D2, and 25(OH)D3) and the dichotomized outcomes of advanced fibrosis (NFS and FIB-4 high-risk categories) was examined using multivariable logistic regression. Three stepwise adjustment models were constructed. Model 1 was unadjusted. Model 2 adjusted for sex, smoking status, alcohol consumption, marital status, season of blood draw, and HbA1c. Model 3 further adjusted for fatty liver, CHD, hypertension, triglycerides, and LDL-C. For analyses using FIB-4 as the outcome, BMI was additionally included in Models 2 and 3, whereas BMI was not included in the NFS models because BMI is already incorporated into the NFS formula. Results are reported as odds ratios (ORs) with 95% confidence intervals (CIs). Furthermore, the dose-response relationship between vitamin D metabolites and the NAFLD Fibrosis Score (NFS) was evaluated using restricted cubic spline (RCS) regression. Non-linearity was formally assessed via the likelihood ratio test, comparing models with linear terms against models incorporating spline terms.

NHANES-specific statistical procedures: Due to the intricate probability sampling design and targeted oversampling employed in NHANES to ensure national representativeness, all analyses utilizing the NHANES data were conducted in accordance with official NHANES analytic guidance. Specifically, appropriate sample weights, clustering, and stratification were applied to account for the complex sampling design. Because six 2-year survey cycles (2007–2018) were combined, the MEC examination weight (WTMEC2YR) was divided by 6 to generate the pooled analytic weight for the merged dataset. In these weighted descriptive analyses, continuous variables are expressed as mean ± standard error of the mean (SEM) and categorical variables as *n* (%); vitamin D measures are presented as median (IQR). Differences were assessed using weighted one-way analysis of variance (ANOVA) for continuous variables and weighted chi-square tests for categorical variables.

Survival and prognostic analysis: Multivariable Cox proportional-hazards models were employed to assess associations of NFS and FIB-4 with all-cause, cardiovascular, and diabetes-related mortality, stratified by vitamin D status; hazard ratios with 95% CIs are reported. Covariates adjusted for in the NFS models were sex, smoking status, alcohol consumption, marital status, HbA1c, ethnicity, education, poverty-income ratio, and hyperlipidemia. For all-cause and cardiovascular mortality models, the FIB-4 models included the same covariates with the addition of BMI. The dose-response relationships between NFS/FIB-4 and vitamin D (total 25(OH)D, 25(OH)D2, 25(OH)D3) were explored using RCS analysis; the best-fitting RCS curve was determined by evaluating different nodes and adjustment positions and selecting the one with the lowest Akaike Information Criterion (AIC) value. Where non-linearity was detected, apparent inflection regions were explored descriptively; these exploratory estimates were not interpreted as clinical cut-offs. Kaplan–Meier curves were used to illustrate survival probabilities by NFS and FIB-4 categories. The proportional hazards assumption was assessed for the main Cox regression models using Schoenfeld residuals (R package survival, function cox.zph); no significant violations were detected, and full results are provided in Supplementary Table S4. Because diabetes-related deaths were sparse in the FIB-4 > 3.25 stratum, the FIB-4–diabetes-related mortality model used a reduced exploratory covariate specification, as detailed in Supplementary Table S4. The primary analyses were performed on complete cases; participants with missing values in variables required for a given model were excluded from that analysis. To assess robustness to missing data, sensitivity analyses using multiple imputation by chained equations (20 imputed datasets) were conducted for the NHANES logistic regression models (Supplementary Methods; Supplementary Table S5), and the results were consistent with the primary analyses. Because participants excluded for missing serum 25(OH)D or fibrosis-score data lacked the key exposure- or outcome-defining variables, a detailed comparison with these participants was not feasible. For the NHANES participants with available serum vitamin D and fibrosis-score data (*n* = 4,616), 4,161 were included in the complete-case covariate-adjusted logistic and Cox regression models, whereas 455 were excluded because of missing model covariates, mainly poverty-income ratio, education level, or marital status. To assess potential selection bias, we compared these two groups with respect to age, sex, BMI, HbA1c, serum 25(OH)D3, and fibrosis-score category; the results are reported in Supplementary Table S10. All statistical analyses were two-tailed, with significance set at *P* < 0.05, and were performed using R version 4.5.2 (R Foundation for Statistical Computing, Vienna, Austria).

### 2.6 Bioinformatics analysis

The Comparative Toxicogenomics Database (CTD) was queried using the disease term “Liver Cirrhosis” (17), which was used as the closest curated CTD disease term representing advanced fibrotic liver disease. From the disease-associated chemicals listed in CTD, Vitamin D was selected, and the CTD pair-specific chemical–disease inference network for Vitamin D and Liver Cirrhosis was examined. CTD integrates curated chemical–gene interactions and gene–disease associations to generate inferred chemical–disease relationships through shared inference-network genes. The database was accessed on 16 June 2026. The current CTD entry identified 14 inference-network genes supporting the Vitamin D–Liver Cirrhosis relationship: ACTA2, ALB, CCN2, CD14, COL1A1, GC, IL6, NFE2L2, SMAD3, SOD2, SPP1, TGFB1, TNF, and VEGFA. For transparency, the Vitamin D chemical–gene set contained 131 unique genes and the independent Liver Cirrhosis disease–gene set contained 24,397 unique genes in the CTD-derived files used during revision. Because the unrestricted CTD Liver Cirrhosis disease–gene set was broad and non-specific, we did not interpret the simple overlap size as a statistical enrichment signal. Instead, the downstream analysis was restricted to the CTD pair-specific Vitamin D–Liver Cirrhosis inference-network genes. Because these candidate genes were extracted from a CTD pair-specific inference network rather than from a *de novo* unrestricted overlap of two independently defined gene sets, no formal hypergeometric or Fisher's exact test was interpreted for the unrestricted overlap. These 14 CTD-derived inference genes were submitted to STRING for exploratory protein–protein interaction (PPI) analysis, with the species restricted to Homo sapiens. No additional interactors were added. To maintain consistency with the CTD-derived candidate gene set, an input-only STRING network was generated, retaining only the 14 CTD inference genes. A high-confidence interaction threshold was applied, defined as STRING combined score ≥0.70. Input genes without retained interactions at this threshold were retained as isolated nodes in the visualization, and non-input STRING nodes were excluded from the final network figure. The interaction table was exported and visualized in R (version 4.2.1) using the igraph (version 1.4.1) and ggraph (version 2.1.0) packages. Centrally connected genes were interpreted descriptively based on network connectivity and interaction strength. Gene Ontology (GO) and Kyoto Encyclopedia of Genes and Genomes (KEGG) enrichment analyses were performed using the CTD Gene Set Analyzer, and enriched terms/pathways with corrected *P* < 0.01 were considered significant. The downstream PPI and enrichment analyses were interpreted as exploratory.

## 3 Results

### 3.1 Baseline characteristics of the study populations

Among individuals with T2DM, those categorized as high-risk for advanced liver fibrosis (NFS > 0.676 or FIB-4 > 3.25) differed significantly from the low-risk group across multiple baseline characteristics (Table 1). The high-risk participants were significantly older, had a lower proportion of males, and reported lower rates of smoking and alcohol consumption, but exhibited a markedly higher prevalence of hypertension. BMI differed significantly between risk groups, particularly in the NFS-defined cohort, where the high-risk group had a higher BMI. Additionally, the high-risk group showed a higher prevalence of CHD, whereas HbA1c levels were significantly lower in the high-risk group. Regarding vitamin D status, total serum 25(OH)D levels were significantly lower in the high-risk group compared to the low-risk group (NFS: 13.6 vs. 14.3 ng/mL, *P* = 0.015; FIB-4: 12.1 vs. 14.2 ng/mL, *P* = 0.035). Serum 25(OH)D3 concentrations were similarly reduced in the high-risk groups (*P* < 0.001 for both scores). Moreover, the distribution of 25(OH)D3 differed across tertiles: a significantly higher percentage of high-risk patients fell into the lowest tertile (NFS: 38.8% vs. 31.1%, *P* = 0.004; FIB-4: 52.1% vs. 32.5%, *P* = 0.002). By contrast, serum 25(OH)D2 levels did not differ significantly between groups (*P* > 0.05).

**Table 1:** Population characteristics by categories of NFS and FIB-4 in T2DM.

Variables	NFS ≤0.676 (*n* = 1432)	NFS >0.676 (*n* = 546)	*P* ^a^	FIB-4 ≤3.25 (*n* = 1905)	FIB-4 >3.25 (*n* = 73)	*P* ^a^
Sex, *n* (%)			0.038			0.014
Male	993 (69.3)	352 (64.5)		1305 (68.5)	40 (54.8)	
Female	439 (30.7)	194 (35.5)		600 (31.5)	33 (45.2)	
Age	55.5 ± 11.7	66.9 ± 9.3	<0.001	58.3 ± 12.1	68.1 ± 10.7	<0.001
Smoke, *n* (%)			0.040			0.225
No	905 (63.2)	372 (68.1)		1225 (64.3)	52 (71.2)	
Yes	527 (36.8)	174 (31.9)		680 (35.7)	21 (28.8)	
Alcohol, *n* (%)			<0.001			0.055
No	1020 (71.2)	441 (80.8)		1400 (73.5)	61 (83.6)	
Yes	412 (28.8)	105 (19.2)		505 (26.5)	12 (16.4)	
Marital status, *n* (%)			<0.001			0.015
Married	1338 (93.4)	510 (93.4)		1783 (93.6)	65 (89)	
Unmarried	46 (3.2)	3 (0.5)		49 (2.6)	0 (0)	
Divorced	24 (1.7)	8 (1.5)		30 (1.6)	2 (2.7)	
Widowed	24 (1.7)	25 (4.6)		43 (2.3)	6 (8.2)	
BMI (kg/m^2^)	25.3 ± 3.6	25.8 ± 3.7	0.006	25.4 ± 3.7	24.4 ± 3.8	0.022
Fatty liver, *n* (%)			<0.001			<0.001
No	953 (66.6)	442 (81)		1,330 (69.8)	65 (89)	
Yes	479 (33.4)	104 (17)		575 (30.2)	8 (10)	
CHD, *n* (%)			<0.001			0.075
No	1212 (84.6)	384 (70.3)		1543 (81)	53 (72.6)	
Yes	220 (15.4)	162 (29.7)		362 (17)	20 (27.4)	
Hypertension, *n* (%)			<0.001			0.031
No	594 (41.5)	159 (29.1)		734 (38.5)	19 (23)	
Yes	838 (58.5)	387 (70.9)		1171 (61.5)	54 (74)	
HbA1c (%)	7.9 ± 1.9	7.4 ± 1.7	<0.001	7.7 ± 1.8	7.3 ± 1.8	0.029
TC (mmol/L)	4.2 ± 1.3	4.0 ± 1.5	0.007	4.2 ± 1.3	3.8 ± 1.6	0.017
LDL-C (mmol/L)	2.4 ± 1.0	2.1 ± 1.1	<0.001	2.3 ± 1.0	1.9 ± 1.0	<0.001
HDL-C (mmol/L)	1.1 ± 0.3	1.1 ± 0.3	0.053	1.1 ± 0.3	1.1 ± 0.3	0.753
Non-HDL-C (mmol/L)	3.2 ± 1.1	2.9 ± 1.3	<0.001	3.1 ± 1.2	2.8 ± 1.4	0.013
Season, *n* (%)			0.049			0.691
Spring	453 (31.6)	207 (37.9)		639 (33.5)	21 (28.8)	
Summer	301 (19)	99 (18.1)		382 (20.1)	18 (24.7)	
Autumn	342 (23.9)	128 (23.4)		454 (23.8)	16 (21.9)	
Winter	336 (23.5)	112 (20.5)		430 (22.6)	18 (24.7)	
25(OH)D (ng/mL)	14.3 (10.0, 20.0)	13.6 (8.9, 19.2)	0.015	14.2 (9.8, 19.7)	12.1 (7.2, 19.8)	0.035
25(OH)D Tertiles, *n* (%)			0.223			0.017
Tertile 1	461 (32.2)	197 (36.1)		623 (32.7)	35 (47.9)	
Tertile 2	481 (33.6)	179 (32.8)		644 (33.8)	16 (21.9)	
Tertile 3	490 (34.2)	170 (31.1)		638 (33.5)	22 (30.1)	
25(OH)D2 (ng/mL)	0.6 (0.2, 1.7)	0.7 (0.3, 2.5)	0.066	0.6 (0.2, 1.7)	0.8 (0.3, 2.8)	0.231
25(OH)D2 Tertiles, *n* (%)			0.172			0.045
Tertile 1	481 (33.6)	173 (31.7)		630 (33.1)	24 (32.9)	
Tertile 2	490 (34.2)	173 (31.7)		647 (31)	16 (21.9)	
Tertile 3	461 (32.2)	200 (36.6)		628 (30)	33 (45.2)	
25(OH)D3 (ng/mL)	12.2(8.0, 17.0)	11.1 (6.3, 15.8)	<0.001	12.0 (7.8, 16.8)	8.5 (5.0, 14.4)	<0.001
25(OH)D3 Tertiles, *n* (%)			0.004			0.002
Tertile 1	445 (31.1)	212 (38.8)		619 (32.5)	38 (52.1)	
Tertile 2	488 (34.1)	173 (31.7)		643 (33.8)	18 (24.7)	
Tertile 3	499 (34.8)	161 (29.5)		643 (33.8)	17 (23.3)	

Data are mean ±SD, median (IQR), or *n* (%). ^a^*P* values were calculated using ANOVA (for normal continuous variables), Kruskal-Wallis test (for skewed continuous variables), or χ^2^ (for categorical variables).

BMI, body mass index; CHD, coronary heart disease; HbA1c, hemoglobin A1c; TC, total cholesterol; LDL-C, low-density lipoprotein cholesterol; HDL-C, high-density lipoprotein cholesterol; Non-HDL-C, non-high-density lipoprotein cholesterol; ANOVA, analysis of variance; 25(OH)D, 25-hydroxyvitamin D; NFS, NAFLD Fibrosis Score (historical name retained for the scoring system); FIB-4, Fibrosis-4 index.

### 3.2 Association of different forms of vitamin D with NFS and FIB-4

In the multivariable analysis (Table 2), total 25(OH)D assessed as a continuous variable was inversely associated with both NFS (OR = 0.98, *P* = 0.002) and FIB-4 (OR = 0.97, *P* = 0.05) after adjusting for confounders including sex, lifestyle factors, season, BMI, and HbA1c. In tertile analyses, participants in the highest 25(OH)D tertile had significantly reduced odds of elevated NFS (OR = 0.77, *P* = 0.039) and FIB-4 (OR = 0.55, *P* = 0.031) compared to the lowest tertile, exhibiting clear dose–response relationships (*P* for trend < 0.05).

**Table 2:** Association between 25(OH)D, 25(OH)D2, 25(OH)D3 and NFS/FIB-4 in multivariable logistic regression models in T2DM.

Variable	Model 1 OR (95% CI)	*P*	Model 2 OR (95% CI)	*P*	Model 3 OR (95% CI)	*P*
Panel A. NFS						
25(OH)D (continuous)	0.98 (0.97, 1)	0.008	0.98 (0.97, 0.99)	0.001	0.98 (0.97, 0.99)	0.002
25(OH)D tertile 1	Reference		Reference		Reference	
25(OH)D tertile 2	0.87 (0.68, 1.10)	0.247	0.85 (0.67, 1.09)	0.199	0.89 (0.69, 1.14)	0.343
25(OH)D tertile 3	0.81 (0.63, 1.03)	0.081	0.74 (0.58, 0.95)	0.019	0.77 (0.59, 0.99)	0.039
Trend test	0.90 (0.80, 1.02)	0.090	0.85 (0.75, 0.97)	0.015	0.87 (0.76, 0.99)	0.037
25(OH)D2 (continuous)	1.01 (0.99, 1.03)	0.250	1.00 (0.98, 1.02)	0.868	1.00 (0.98, 1.01)	0.641
25(OH)D3 (continuous)	0.97 (0.96, 0.99)	<0.001	0.98 (0.96, 0.99)	0.001	0.98 (0.96, 0.99)	0.002
25(OH)D3 tertile 1	Reference		Reference		Reference	
25(OH)D3 tertile 2	0.74 (0.58, 0.94)	0.013	0.77 (0.60, 0.98)	0.031	0.81 (0.63, 1.05)	0.088
25(OH)D3 tertile 3	0.68 (0.53, 0.86)	0.002	0.68 (0.53, 0.88)	0.004	0.74 (0.57, 0.94)	0.019
Trend test	0.82 (0.73, 0.93)	0.001	0.82 (0.72, 0.94)	0.003	0.85 (0.74, 0.97)	0.016
Panel B. FIB-4						
25(OH)D (continuous)	0.97 (0.94, 1.01)	0.114	0.97 (0.94, 1.00)	0.057	0.97 (0.94, 1.00)	0.050
25(OH)D tertile 1	Reference		Reference		Reference	
25(OH)D tertile 2	0.41 (0.22, 0.74)	0.030	0.41 (0.22, 0.75)	0.003	0.43 (0.23, 0.79)	0.006
25(OH)D tertile 3	0.60 (0.35, 1.03)	0.063	0.56 (0.32, 0.96)	0.034	0.55 (0.31, 0.95)	0.031
Trend test	0.75 (0.56, 1.01)	0.058	0.71 (0.53, 0.96)	0.024	0.70 (0.52, 0.95)	0.022
25(OH)D2 (continuous)	1.02 (0.98, 1.05)	0.244	1.01 (0.96, 1.04)	0.780	1.00 (0.96, 1.04)	0.896
25(OH)D3 (continuous)	0.95 (0.92, 0.99)	0.010	0.96 (0.93, 0.99)	0.025	0.95 (0.92, 0.99)	0.028
25(OH)D3 tertile 1	Reference		Reference		Reference	
25(OH)D3 tertile 2	0.47 (0.26, 0.81)	0.006	0.48 (0.28, 0.87)	0.015	0.50 (0.28, 0.90)	0.025
25(OH)D3 tertile 3	0.44 (0.24, 0.77)	0.004	0.48 (0.26, 0.85)	0.012	0.48 (0.26, 0.86)	0.013
Trend test	0.63 (0.46, 0.85)	0.002	0.64 (0.47, 0.87)	0.004	0.64 (0.46, 0.88)	0.005

OR, odds ratio; CI, confidence interval; 25(OH)D, serum 25-hydroxyvitamin D; 25(OH)D2, serum 25-hydroxyvitamin D2; 25(OH)D3, serum 25-hydroxyvitamin D3; FIB-4, Fibrosis-4 index; NFS, NAFLD Fibrosis Score (historical name retained for the scoring system); CHD, coronary heart disease; LDL-C, low-density lipoprotein cholesterol.

Model 1 was unadjusted.

Model 2 was adjusted for sex, smoking status, alcohol consumption, marital status, season, and HbA1c.

Model 3 was further adjusted for fatty liver, CHD, hypertension, triglycerides, and LDL-C.

BMI was included as a covariate in the analyses for FIB-4 (Models 2^e^ and 3^f^) but was excluded from the analyses for NFS (Models 2^b^ and 3^c^) to avoid collinearity.

Similarly, 25(OH)D3 showed an inverse association with fibrosis risk. The highest tertile of 25(OH)D3 was associated with lower odds of elevated NFS (OR = 0.74, *P* = 0.019) and FIB-4 (OR = 0.48, *P* = 0.013), with significant trends. Conversely, no significant associations were observed between 25(OH)D2 and fibrosis risk in any multivariable model, suggesting that the overall association of total 25(OH)D was largely driven by the 25(OH)D3 isoform. Although the univariate distribution of 25(OH)D2 tertiles differed across FIB-4 categories in Table 1 (*P* = 0.045), this apparent association was attenuated to non-significance after multivariable adjustment (Table 2), most likely reflecting confounding by age and comorbidity burden, which are strongly associated with both ergocalciferol exposure and fibrosis-score category.

### 3.3 Characterization of dose-response relationships

**Figure 1:**
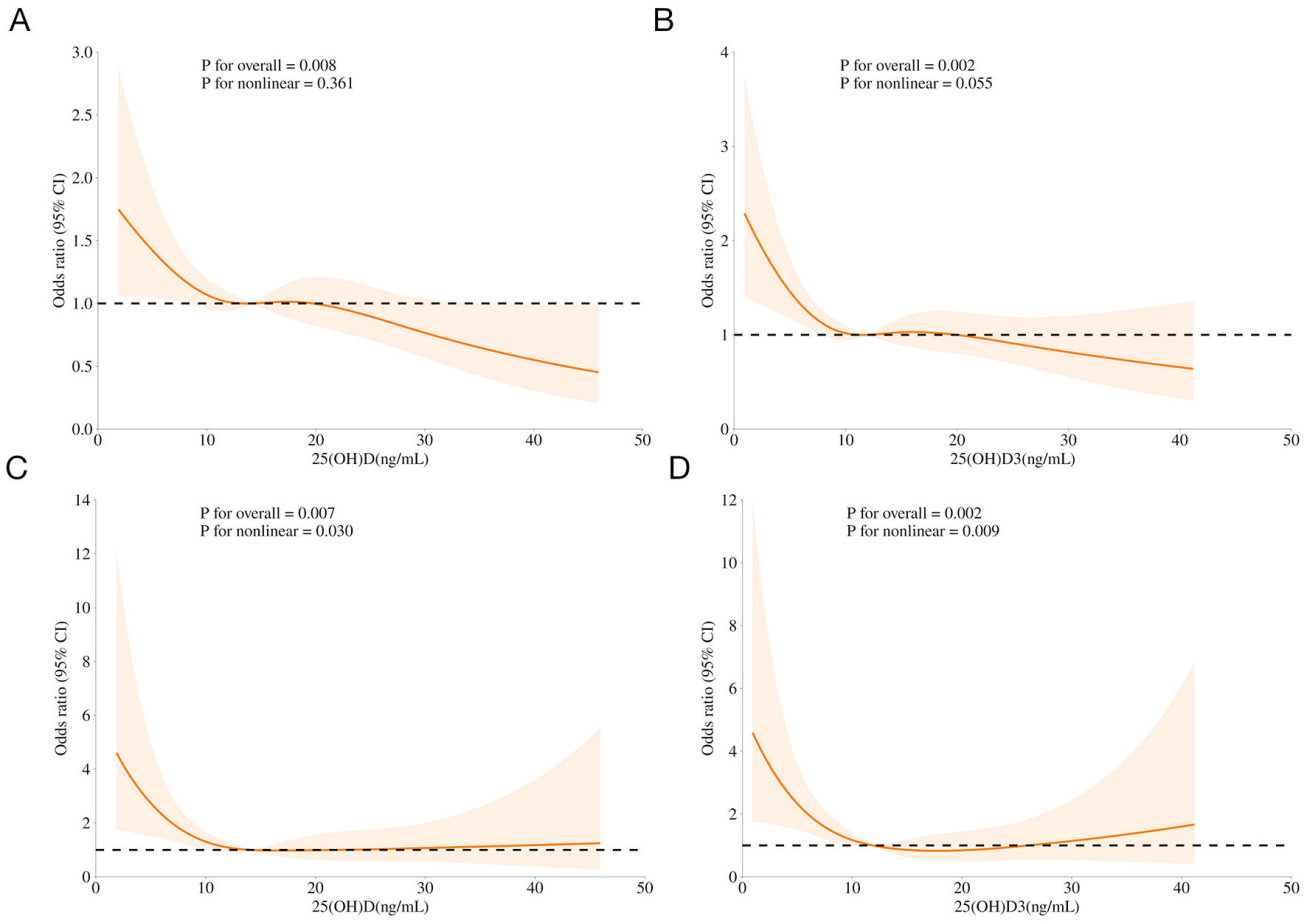
Restricted cubic spline (RCS) analyses of the dose-response associations between serum vitamin D forms and the risk of advanced liver fibrosis in the internal hospital population. **(A, B)** Associations of serum total 25(OH)D **(A)** and 25(OH)D3 **(B)** with the risk of advanced fibrosis assessed by the NFS score. **(C, D)** Associations of serum total 25(OH)D **(C)** and 25(OH)D3 **(D)** with the risk of advanced fibrosis assessed by the FIB-4 index.

Restricted cubic spline (RCS) analyses further characterized these patterns in the internal hospital cohort (Figure 1). Total 25(OH)D showed a largely linear inverse association with NFS, whereas its association with FIB-4 was non-linear (P-nonlinearity = 0.030), with an RCS-derived apparent inflection point at 14.14 ng/mL (35.3 nmol/L). Similarly, 25(OH)D3 showed a non-linear association with FIB-4 (P-nonlinearity = 0.009), with an apparent inflection point at 11.88 ng/mL (29.66 nmol/L). These findings suggest that the inverse association between 25(OH)D3 and advanced fibrosis may plateau around this RCS-derived range; however, these values were descriptive estimates and were not intended as clinical cut-offs.

### 3.4 Validation in the NHANES cohort

Using NHANES data (Table 3), we validated the associations. While continuous total 25(OH)D showed no significant association with fibrosis scores, categorical analyses revealed an inverse association in the middle tertile (T2) for both NFS and FIB-4 (*P* < 0.05). When analyzing specific isoforms, the results were generally consistent with the internal cohort findings. 25(OH)D3 showed an inverse association with fibrosis risk. Unlike total 25(OH)D, the association of 25(OH)D3 remained significant in the highest tertile (NFS: OR = 0.654, *P* = 0.011; FIB-4: OR = 0.499, *P* = 0.014). By contrast, elevated 25(OH)D2 (T3) was associated with increased NFS risk (OR = 1.405, *P* = 0.003). A multiple-imputation sensitivity analysis for selected missing NHANES covariates yielded directionally consistent results with the primary complete-case analyses (Supplementary Table S5). Compared with participants included in the complete-case covariate-adjusted analyses (*n* = 4,161), those excluded because of missing model covariates (*n* = 455) were older and had a higher prevalence of NFS-defined advanced fibrosis. BMI, HbA1c, serum 25(OH)D3, sex distribution, and FIB-4-defined advanced fibrosis did not differ significantly between groups (Supplementary Table S10). Subgroup sensitivity analyses stratified by age and BMI category showed generally directionally similar associations between 25(OH)D3 and advanced fibrosis. The inverse association remained statistically significant for NFS-defined advanced fibrosis among participants aged ≥60 years, whereas estimates in other subgroups were directionally consistent but less precise, with wider confidence intervals due to reduced subgroup sample sizes (Supplementary Table S6). After excluding participants in the highest decile of fibrosis scores, the inverse association between 25(OH)D3 and NFS-defined advanced fibrosis remained evident, whereas the corresponding FIB-4-based estimate was attenuated toward the null (Supplementary Table S7).

**Table 3:** ORs (95% CIs) for NFS and FIB-4 according to vitamin D metabolites in the NHANES cohort.

Variables	NFS OR (95% CI)	*P* value	FIB-4 OR (95% CI)	*P* value
25 (OH)D (continuous)	0.998 (0.994, 1.002)	0.264	1.000 (0.991, 1.008)	0.911
25 (OH)D tertiles				
Tertile 1	Reference		Reference	
Tertile 2	0.733 (0.543, 0.990)	0.043	0.472 (0.237, 0.943)	0.034
Tertile 3	0.829 (0.601, 1.144)	0.251	0.661 (0.370, 1.181)	0.159
25 (OH)D2 (continuous)	1.009 (1.003, 1.014)	0.004	1.008 (0.999, 1.016)	0.080
25 (OH)D2 tertiles				
Tertile 1	Reference		Reference	
Tertile 2	1.104 (0.879, 1.386)	0.389	0.702 (0.404, 1.218)	0.205
Tertile 3	1.405 (1.129, 1.749)	0.003	1.191 (0.666, 2.127)	0.551
25 (OH)D3 (continuous)	0.995 (0.991, 0.999)	0.026	0.997 (0.988, 1.006)	0.493
25 (OH)D3 tertiles				
Tertile 1	Reference		Reference	
Tertile 2	0.639 (0.471, 0.868)	0.005	0.356 (0.186, 0.683)	0.002
Tertile 3	0.654 (0.472, 0.906)	0.011	0.499 (0.288, 0.863)	0.014

OR, odds ratio; CI, confidence interval.

NFS model was adjusted for sex, smoking status, alcohol consumption, marital status, HbA1c, race/ethnicity, education level, poverty-income ratio, and hyperlipidemia. FIB-4 model was adjusted for the same covariates plus BMI. Analyses were performed using complete cases; multiple-imputation sensitivity analyses are reported in Supplementary Table S5.

**Figure 2:**
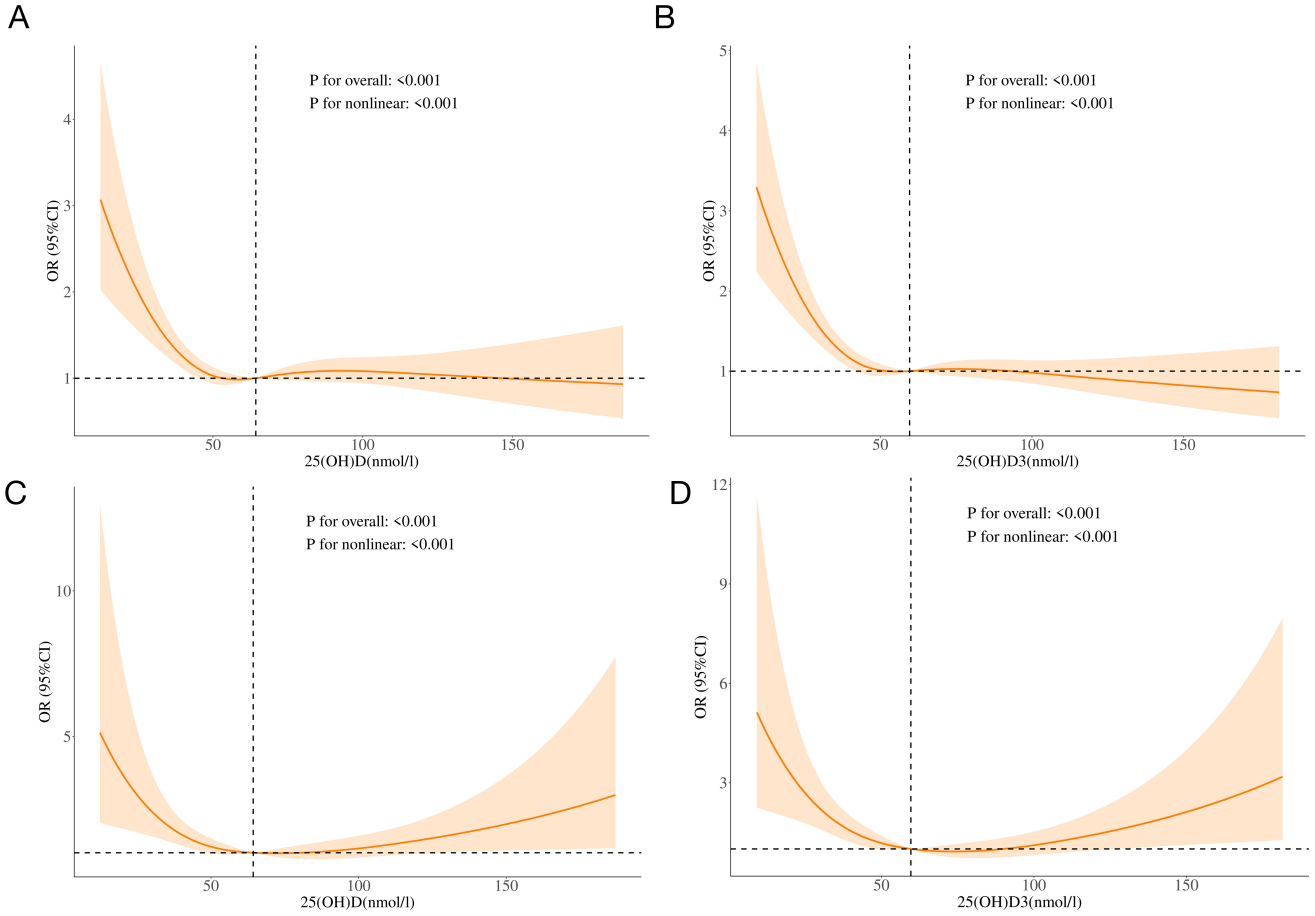
Restricted cubic spline (RCS) analyses of the dose-response associations between serum vitamin D forms and the risk of advanced liver fibrosis in the NHANES population. **(A, B)** Associations of serum total 25(OH)D **(A)** and 25(OH)D3 **(B)** with the risk of advanced fibrosis assessed by the NFS score. **(C, D)** Associations of serum total 25(OH)D **(C)** and 25(OH)D3 **(D)** with the risk of advanced fibrosis assessed by the FIB-4 index.

RCS analyses in NHANES (Figure 2) confirmed significant non-linear relationships. For NFS, total 25(OH)D showed an apparent inflection point at 25.72 ng/mL (64.3 nmol/L), while 25(OH)D3 followed a U-shaped pattern with an apparent inflection point at 23.86 ng/mL (59.65 nmol/L). Similar non-linear trends were observed for FIB-4. Of note, for FIB-4 in NHANES the per-unit continuous association of 25(OH)D3 was not statistically significant (OR = 0.997, *P* = 0.493), whereas the tertile contrasts were significant (Tertile 2 vs. Tertile 1, OR = 0.356, *P* = 0.002; Tertile 3 vs. Tertile 1, OR = 0.499, *P* = 0.014). This pattern is consistent with a non-linear or threshold-type relationship, and may also reflect the greater statistical power of contrasting extreme tertiles relative to estimating a per-unit linear slope; it should therefore not be interpreted as a contradictory finding.

### 3.5 Survival analysis and association of NFS/FIB-4 with all-cause, cardiovascular and diabetes mortality

**Figure 3:**
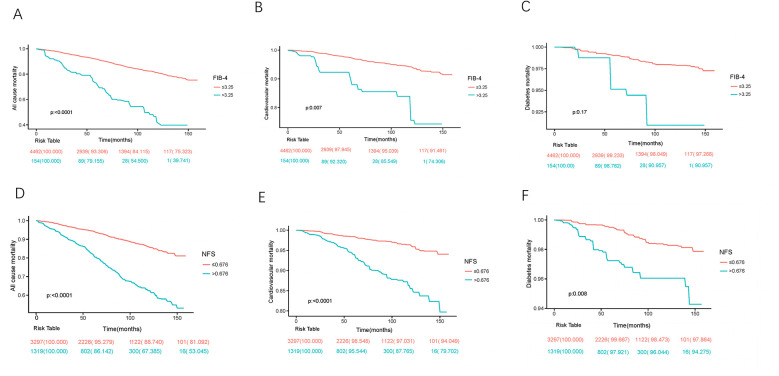
Kaplan–Meier survival curves for all-cause and cause-specific mortality stratified by liver fibrosis scores. **(A–C)** Survival probability stratified by the NFS score for all-cause mortality **(A)**, cardiovascular mortality **(B)**, and diabetes-related mortality **(C)**. **(D–F)** Survival probability stratified by the FIB-4 index for all-cause mortality **(D)**, cardiovascular mortality **(E)**, and diabetes-related mortality **(F)**.

**Figure 4:**
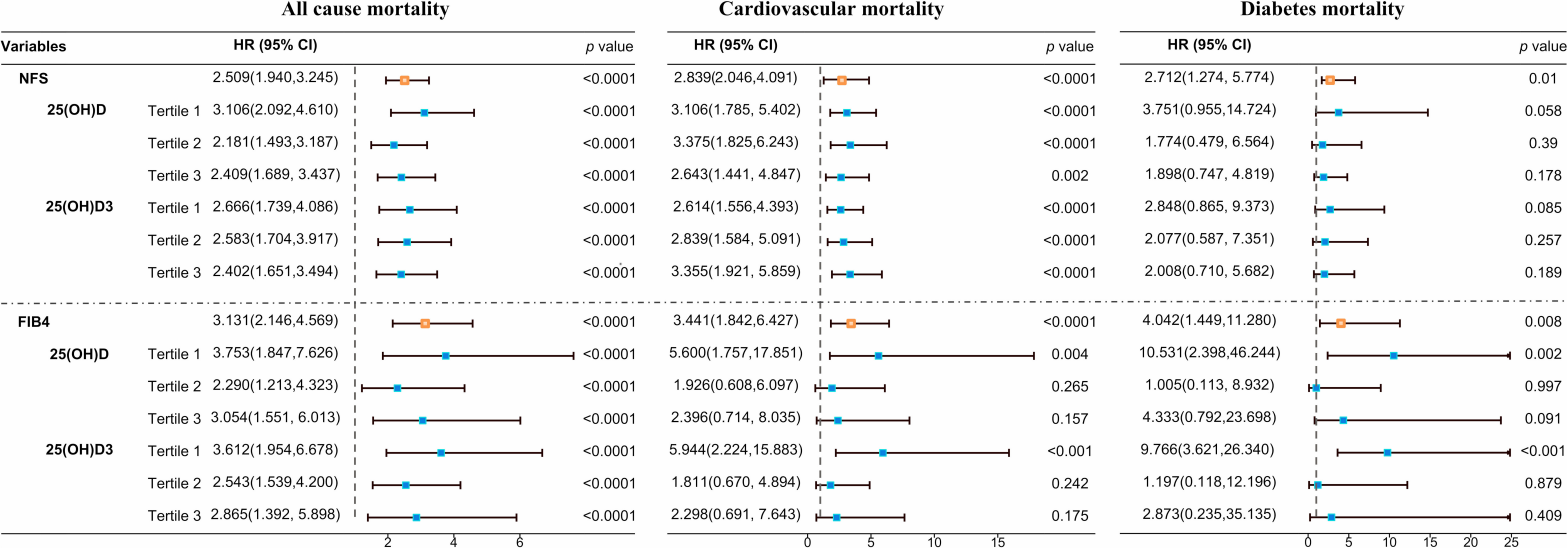
Forest plot illustrating the multivariable-adjusted hazard ratios (HRs) and 95% confidence intervals (CIs) for the associations between advanced liver fibrosis (NFS/FIB-4) and all-cause, cardiovascular, and diabetes-related mortality, stratified by tertiles of total 25(OH)D and 25(OH)D3.

Kaplan–Meier analysis showed that participants with high-risk NFS and FIB-4 scores had lower survival for all-cause and cause-specific mortality (Figure 3). In multivariable Cox proportional hazards models adjusted for confounders, both elevated NFS and FIB-4 were associated with increased mortality risks (Figure 4; Supplementary Table S2). Specifically, high-risk FIB-4 scores predicted all-cause (HR = 3.131, *P* < 0.0001), cardiovascular (HR = 3.441, *P* < 0.0001), and diabetes-related mortality (HR = 4.042, *P* = 0.008); the diabetes-related estimate was based on a limited number of events and should be interpreted with the caution detailed below. Schoenfeld residual tests did not indicate significant violations of the proportional hazards assumption in the main Cox models (Supplementary Table S4).

Stratified analyses showed that these mortality risks varied according to vitamin D status (Supplementary Table S3). Among participants with high fibrosis risk (FIB-4 > 3.25), those in the lowest 25(OH)D3 tertile (Tertile 1) showed the highest point estimates for cause-specific mortality. The estimate for cardiovascular mortality remained elevated (HR = 5.944, 95% CI: 2.224–15.883) and was based on a larger number of events. By contrast, the diabetes-related mortality estimate in the FIB-4 high-risk/low-25(OH)D3 subgroup was based on only five events and was therefore treated as exploratory; detailed point estimates are provided in Supplementary Table S3. As 25(OH)D3 levels increased to the highest tertile (Tertile 3), the associations with cardiovascular and diabetes-related mortality became statistically non-significant (*P* = 0.175 and *P* = 0.409, respectively). In contrast, the association with all-cause mortality, while attenuated from Tertile 1 (HR = 3.612) to Tertile 3 (HR = 2.865), remained statistically significant (*P* < 0.0001). Because diabetes-related deaths were limited among participants with FIB-4 > 3.25, particularly after stratification by 25(OH)D3 tertiles, the corresponding HR estimates should be interpreted cautiously. Among participants with FIB-4 > 3.25, only 5 diabetes-related deaths were observed, including 3, 1, and 1 events across increasing 25(OH)D3 tertiles (Supplementary Table S8). Because this exploratory model included multiple covariates but was based on only 5 events, it is severely underpowered by conventional criteria (which suggest approximately 10 events per covariate); the corresponding hazard ratio is statistically unstable and should not be interpreted as a reliable effect-size estimate.

### 3.6 Potential molecular mechanisms linking vitamin D to liver fibrosis

**Figure 5:**
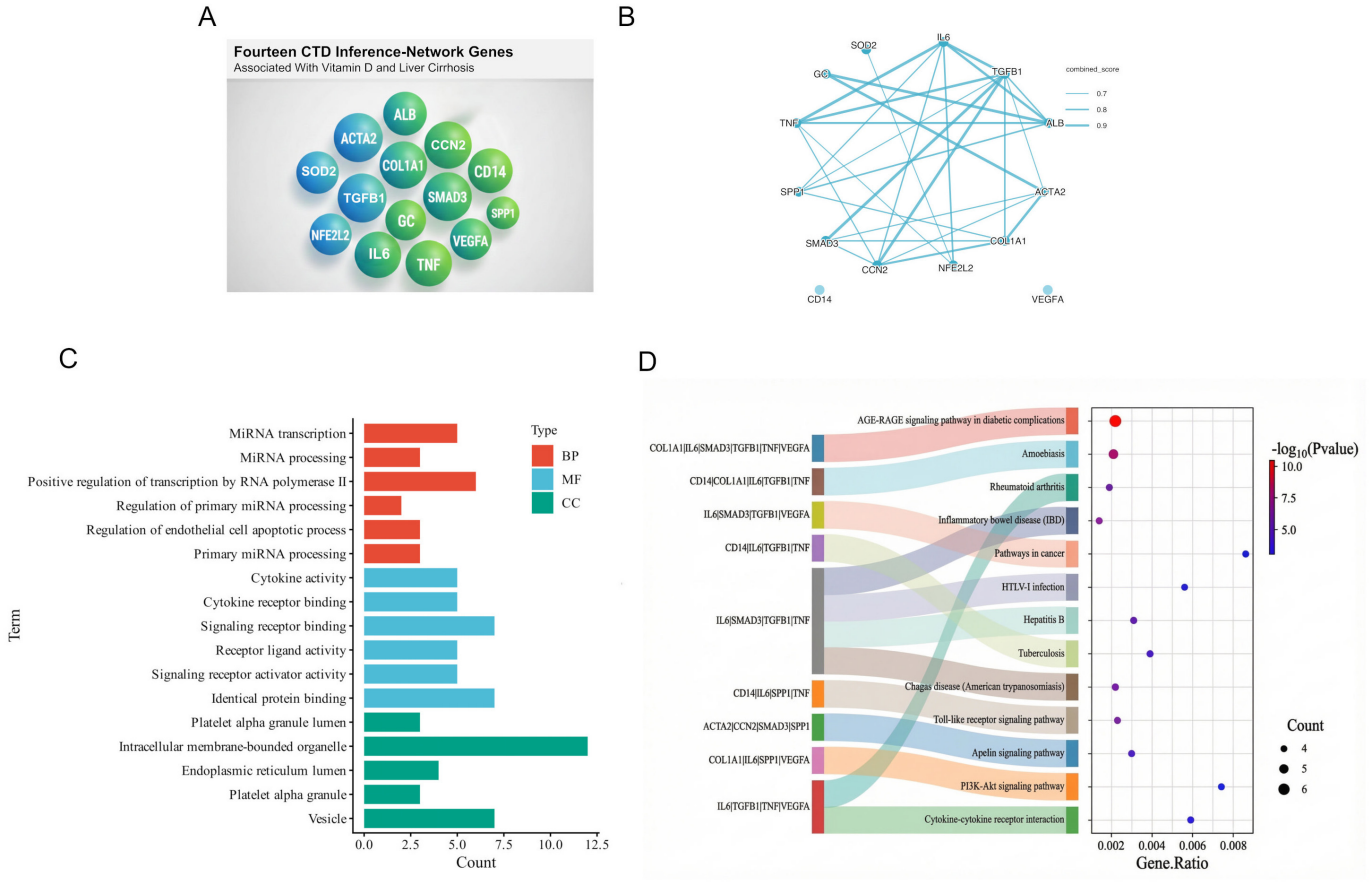
CTD-derived Vitamin D–Liver Cirrhosis inference genes and exploratory pathway analysis. **(A)** Fourteen CTD pair-specific inference-network genes supporting the Vitamin D–Liver Cirrhosis relationship. **(B)** Exploratory STRING input-only protein-protein interaction network restricted to the 14 CTD-derived inference genes. Edges represent STRING protein-protein associations retained at a high-confidence threshold, defined as combined score ≥0.70. CD14 and VEGFA were retained as isolated input nodes because they had no retained interactions at this threshold. No additional STRING interactors were added. **(C)** Gene Ontology enrichment analysis. **(D)** KEGG pathway enrichment analysis. PPI, protein-protein interaction; GO, Gene Ontology; KEGG, Kyoto Encyclopedia of Genes and Genomes; CTD, Comparative Toxicogenomics Database.

In CTD, Vitamin D was listed among chemicals associated with Liver Cirrhosis, with direct therapeutic evidence, an inference score of 25.60, and 24 supporting references. The pair-specific CTD Vitamin D—Liver Cirrhosis inference network contained 14 genes (Figure 5A): ACTA2, ALB, CCN2, CD14, COL1A1, GC, IL6, NFE2L2, SMAD3, SOD2, SPP1, TGFB1, TNF, and VEGFA. Exploratory STRING analysis was then performed using an input-only network restricted to these 14 genes and a high-confidence threshold of combined score ≥0.70. CD14 and VEGFA were retained as isolated input nodes because they had no retained interactions at this threshold, and no additional STRING interactors were added (Figure 5B).

This input-only high-confidence network retained centrally connected inflammation-, oxidative-stress-, angiogenesis-, and extracellular-matrix-remodeling-related nodes, including IL6, TNF, TGFB1, SOD2, COL1A1, ACTA2, SMAD3, and NFE2L2. GO and KEGG enrichment analyses highlighted pathways related to inflammatory signaling, oxidative stress, extracellular-matrix organization, AGE–RAGE signaling, and PI3K–Akt signaling (Figures 5C, D). Because the CTD candidates were derived from a pair-specific inference network rather than an unrestricted two-list overlap, these bioinformatic findings were interpreted as hypothesis-generating rather than as formal overlap-enrichment evidence.

## 4 Discussion

This dual-population study, conducted in Chinese patients with T2DM and US adults with T2DM from NHANES, examined the distinct associations of vitamin D metabolites with liver fibrosis assessed under the NAFLD framework. In both cohorts, lower serum 25(OH)D3 was consistently and non-linearly associated with advanced fibrosis, whereas 25(OH)D2 showed no inverse association; in NHANES, higher 25(OH)D2 was additionally associated with a modest increase in NFS-defined fibrosis risk. In longitudinal analyses, low 25(OH)D3 was also associated with higher cardiovascular mortality among participants with advanced fibrosis; a similar direction was observed for diabetes-related mortality, although that estimate was based on sparse events and is considered exploratory. Given the observational design, these findings should be interpreted as associations rather than causal effects. Overall, they suggest that isoform-specific assessment, particularly of 25(OH)D3, may provide additional value beyond total vitamin D for risk stratification (18–20).

Because circulating 25(OH)D2 largely reflects ergocalciferol intake or supplementation, the positive association between high 25(OH)D2 and NFS-defined fibrosis in NHANES may reflect confounding by indication rather than a biological harmful effect. Individuals receiving ergocalciferol may have greater baseline comorbidity, malabsorption risk, poorer nutritional status, or physician-recognized vitamin D deficiency. Detailed information on ergocalciferol dose, duration, and clinical indication was not available. Critically, NHANES does not distinguish prescription-dose ergocalciferol—used to treat diagnosed severe vitamin D deficiency, which may itself co-occur with hepatic dysfunction or malabsorption—from dietary ergocalciferol of fungal origin. Because these two sources carry entirely different confounding structures, the 25(OH)D2 finding cannot be interpreted in either direction, as harmful or as neutral, without this information. We therefore refrain from any mechanistic interpretation of the 25(OH)D2 association and note only that it did not show the inverse pattern observed for 25(OH)D3.

However, these values should not be interpreted as universal clinical cut-offs. Rather, they are better viewed as cohort-specific, RCS-derived apparent inflection points that may mark the range at which the association appears to plateau. These RCS-derived values were exploratory descriptive estimates from observational spline models. They were not pre-specified, were not derived from formal changepoint modeling, and should not be interpreted as clinical targets or treatment thresholds. The roughly two-fold difference between the cohort-specific inflection points (approximately 12 ng/mL in the Chinese cohort vs. approximately 24 ng/mL in NHANES) may have several plausible contributors (21, 24). These include differences in laboratory platform, calibration materials, and C3-epimer handling between the two settings; racial and ethnic differences in vitamin D-binding protein affinity and bioavailability; differences in dietary intake and sun-exposure patterns; differences in the covariate adjustment sets available in each cohort (notably the absence of seasonal adjustment in NHANES and the absence of race/ethnicity in the Chinese cohort); and possible differences in the underlying distribution of fibrosis severity between the two populations (22, 23). These factors should be considered when comparing the two inflection estimates, which we regard as exploratory and cohort-specific rather than directly comparable thresholds.

Beyond cross-sectional associations, our study also suggests a prognostic interplay between liver fibrosis and vitamin D status. Among participants with advanced fibrosis, lower 25(OH)D3 was associated with higher cardiovascular mortality, whereas these excess risks were attenuated and no longer statistically significant at higher 25(OH)D3 levels. A similar direction was observed for diabetes-related mortality; however, because the diabetes-related mortality model in the FIB-4 high-risk stratum was based on only 5 events, this estimate is severely underpowered and statistically unstable, and we therefore do not interpret its magnitude as a reliable effect size. By contrast, the association with all-cause mortality remained significant even at higher 25(OH)D3 levels, although the magnitude of risk was reduced. This pattern suggests that vitamin D status may be particularly relevant to metabolic and cardiovascular risk stratification within the fibrosis-positive subgroup, whereas the broader mortality burden associated with advanced liver disease is likely to reflect additional mechanisms not fully captured by vitamin D status alone (25–28). These observations require confirmation in prospective and interventional studies.

Bioinformatic analyses implicated the AGE–RAGE and PI3K–Akt pathways as candidate mechanisms linking vitamin D status to fibrosis-related processes in T2DM. In the hyperglycaemic milieu, accumulation of advanced glycation end products may promote oxidative stress, inflammatory signaling, and hepatic stellate cell activation through RAGE-related pathways (29–31). The enrichment of AGE–RAGE, PI3K–Akt, and cytokine-related signaling pathways, together with the identification of fibrosis- and inflammation-related network nodes, provides a biologically plausible framework connecting altered vitamin D signaling to fibrogenic and inflammatory processes (32–34). However, these pathway-level findings should be regarded as hypothesis-generating rather than confirmatory, and direct mechanistic studies will be required to determine whether altered vitamin D signaling modulates fibrosis progression through these pathways. The PPI findings were based on an input-only high-confidence STRING network restricted to the CTD-derived inference genes and were interpreted descriptively rather than as confirmatory mechanistic evidence.

The primary strengths of this study include the use of LC–MS/MS-based isoform-specific metabolite quantification and validation across two independent, multi-ethnic cohorts. Several limitations should nevertheless be acknowledged. First, the cross-sectional nature of the primary analyses precludes causal inference, and reverse causality is an important concern. More advanced liver fibrosis may impair hepatic 25-hydroxylation of vitamin D through altered CYP2R1/CYP27A1 activity and may reduce albumin-mediated vitamin D transport, thereby lowering circulating 25(OH)D3 as a consequence of disease severity rather than as an upstream determinant. Sensitivity analyses excluding participants in the highest decile of fibrosis scores suggested that the NFS-based association was not driven solely by extreme fibrosis-score values, although the FIB-4-based estimate was attenuated fully to the null (OR = 1.000, *P* = 0.912). This divergence between the two scores deserves comment. FIB-4 incorporates age as a direct multiplicative term, so elevated FIB-4 values in older individuals may partly reflect age rather than true fibrotic burden; after excluding the most extreme FIB-4 values, the residual association with 25(OH)D3 may therefore be diminished. In addition, NFS and FIB-4 incorporate different component variables and may capture partly distinct patient phenotypes in T2DM. Accordingly, the NFS- and FIB-4-based associations should not be regarded as equivalent, and this discordance tempers the strength of any causal interpretation. Therefore, the observed associations should not be interpreted as evidence that low 25(OH)D3 causes fibrosis progression. Second, although NFS and FIB-4 are well-validated non-invasive screening tools, they do not provide the histological resolution of liver biopsy. Their diagnostic performance is also lower in individuals with obesity and in patients younger than 60 years, which may introduce non-differential misclassification of fibrosis stage. To address this concern, we performed subgroup sensitivity analyses stratified by age and BMI category; these analyses showed generally directionally similar estimates, but some subgroup-specific associations were imprecise because of reduced sample size. Third, despite extensive multivariable adjustment, several potentially relevant confounders were not fully captured. In the Chinese hospital cohort, sun exposure (ultraviolet-B dose), dietary vitamin D intake, oily-fish consumption, use of over-the-counter vitamin D supplements, and habitual physical activity were not directly measured. In NHANES, socioeconomic status was only partially captured through the poverty-income ratio and educational attainment, while occupation and neighborhood-level factors were unavailable. In addition, the two cohorts differed in the covariates available for adjustment: the Chinese cohort incorporated season of blood draw but lacked race/ethnicity, poverty-income ratio, and education, whereas NHANES included race/ethnicity, poverty-income ratio, and education but did not incorporate seasonal adjustment. A structured comparison of covariate adjustment between the two cohorts is provided in Supplementary Table S9. These asymmetries may have contributed to the numerically different inflection estimates observed between cohorts, and residual confounding cannot be excluded. In addition, participants excluded from complete-case model analyses because of missing model covariates were older and more likely to have NFS-defined advanced fibrosis than included participants, so selection bias related to missing covariate data cannot be fully excluded. Finally, prospective and interventional studies incorporating histology or validated imaging-based fibrosis assessment, including transient elastography and magnetic resonance elastography, are needed to confirm these population-specific findings and to clarify their clinical relevance.

## 5 Conclusion

This multi-population study reports differential associations of 25(OH)D2 and 25(OH)D3 with advanced liver fibrosis assessed by NFS and FIB-4 in T2DM. Lower 25(OH)D3, but not 25(OH)D2, was associated with advanced fibrosis after multivariable adjustment. Among NHANES participants with advanced fibrosis, low 25(OH)D3 was also associated with higher cardiovascular mortality; the corresponding diabetes-related mortality estimate was based on sparse events and is considered exploratory. Bioinformatic analyses suggested candidate pathways potentially linking vitamin D status to fibrosis-related processes. These findings may support further investigation of isoform-specific 25(OH)D3 assessment for risk stratification in T2DM.

## Data Availability

The NHANES datasets analyzed in this study are publicly available at: https://wwwn.cdc.gov/nchs/nhanes/continuousnhanes/labmethods.aspx?BeginYear=2007. The internal hospital cohort data are not publicly available due to institutional privacy and ethical restrictions, but may be made available from the corresponding author upon reasonable request and with appropriate approvals.
