# Supplementary material for: Serum 25(OH)D3 and advanced liver fibrosis in type 2 diabetes: a Chinese hospital cohort with NHANES validation and mortality follow-up
Source: Front Nutr. 2026 Jul 22;13:1820737. doi: 10.3389/fnut.2026.1820737 (PMC13438427; doi:10.3389/fnut.2026.1820737)
Supplement: Supplementary file 1 [file Supplementary_file_1.docx]

**Supplementary Materials**

## Serum 25(OH)D3 and Advanced Liver Fibrosis in Type 2 Diabetes: A Chinese Hospital Cohort With NHANES Validation and Mortality Follow-up

Guohong Zhao^#1^, Jiaoqian Yang^#1^, Li Zhang^#1^, Shuo Yang^1^, Duolin Niu^1^, Meitong Yang^1^, Zhaowei Gao^2^, Fenghui Ma^3^, Bin Gao^*1^

^1^Department of Endocrinology, Tangdu Hospital, The Fourth Military Medical University, Xi’an 710038, Shaanxi Province, P. R. China.

^2^Department of Clinical laboratory, Tangdu Hospital, The Fourth Military Medical University, Xi’an 710038, Shaanxi Province, P. R. China.

^3^Department of Health Management, Tangdu Hospital, The Fourth Military Medical University, Xi’an 710038, Shaanxi Province, P. R. China.

\# Guohong Zhao, Jiaoqian Yang and Li Zhang contributed equally to this work.

***Corresponding author:**

Bin Gao, Department of Endocrinology, Tangdu Hospital, The Fourth Military Medical University, Xi’an, Shaanxi Province, P. R. China. E-mail: bingao0726@163.com, Tel.: + 86 13772100470; Fax: +86-29-84777730.

**Supplementary Methods**

**Covariates**

For the internal hospital cohort, covariates included sociodemographic factors (sex and marital status), lifestyle indicators (alcohol consumption and smoking status), anthropometric measures (body mass index, BMI), and clinical/metabolic variables including glycated haemoglobin (HbA1c), triglycerides (TG), low-density lipoprotein cholesterol (LDL-C), fatty liver, coronary heart disease (CHD), and hypertension. Season of examination in Shaanxi Province, China, was categorized as spring (March-May), summer (June-August), autumn (September-November), and winter (December-February).

For the NHANES (2007-2018 cycles) cohort, covariates included standard demographic indicators such as age, sex, race/ethnicity (Mexican American/non-Hispanic Black/non-Hispanic White/other), education level (high school or below, high school, college or above), household income-to-poverty ratio, and marital status (married/living with partner/divorced/never married/separated/widowed), as well as lifestyle indicators including alcohol consumption and smoking status, disease status (hyperlipidemia), anthropometric measures (BMI), and glycated haemoglobin (HbA1c).

**NHANES weighting and model specifications**

For NHANES analyses, appropriate sample weights, clustering, and stratification were applied in accordance with NHANES analytic guidance. Because six 2-year survey cycles (2007-2018) were combined, the MEC examination weight (WTMEC2YR) was divided by 6 to generate the pooled analytic weight for the merged dataset. Weighted descriptive analyses, multivariable logistic regression, restricted cubic spline analyses, and Cox proportional-hazards models were conducted within the NHANES analytic framework, with participants missing variables required for a given model excluded from that analysis.

Missing-data handling and sensitivity analysis

Missing data were not imputed in the primary analyses. Participants with missing values in variables required for a given model were excluded from that analysis. Among the 4,616 NHANES participants with available serum vitamin D and fibrosis-score data, 4,161 were included in the complete-case covariate-adjusted analyses, whereas 455 were excluded because of missing model covariates, mainly poverty-income ratio, education level, or marital status. A comparison of included and excluded participants is provided in Table S10. As a sensitivity analysis, missing covariates (marital status, education level, and poverty-income ratio) were imputed using multiple imputation by chained equations, generating 20 imputed datasets. Multinomial logistic regression was used for marital status and education level, and predictive mean matching was used for poverty-income ratio. Only the missing covariates were imputed; outcome variables, exposure variables, and covariates without missingness were not imputed. The principal regression models were then re-fitted in each imputed dataset, and pooled estimates were obtained in R version 4.5.2 using standard multiple-imputation combining procedures.

**Assessment of the proportional hazards assumption**

The proportional hazards assumption was assessed for the main Cox regression models using Schoenfeld residuals, implemented via the cox.zph function of the survival package in R, based on the corresponding covariate-adjusted model specifications used in the primary survival analyses. Tests were performed for both individual covariates and the global model. No significant violations were detected (all global P > 0.30; all main exposure terms P > 0.10). Summary results and the reduced exploratory covariate specification used for the FIB-4–diabetes-related mortality model are provided in Table S4.

**Figure Legends**

**Figure S1. Vitamin D metabolism from precursors to active hormone and downstream signalling.** Vitamin D2 (ergocalciferol) is derived from plant/fungal ergosterol via UV-B (290-315 nm) photoconversion, while vitamin D3 (cholecalciferol) originates from cutaneous photoconversion of 7-dehydrocholesterol and from dietary sources (oily fish, eggs, and dairy products). Both forms undergo hepatic 25-hydroxylation by CYP2R1/CYP27A1 to yield the two major circulating metabolites, 25(OH)D2 and 25(OH)D3, which were quantified in this study by LC-MS/MS. Compared with 25(OH)D2, 25(OH)D3 shows greater affinity for vitamin D-binding protein (DBP), contributing to a longer circulating half-life. Renal 1α-hydroxylation (CYP27B1) generates 1,25-dihydroxyvitamin D [1,25(OH)2D], which mediates pleiotropic effects via the vitamin D receptor (VDR/RXR) and retinoic acid-related orphan receptors (RORα/γ), regulating calcium/phosphate homeostasis, immunity, inflammation, cellular proliferation, and differentiation. CYP24A1 inactivates both 25(OH)D and 1,25(OH)2D via 24-hydroxylation to 24,25(OH)2D for biliary and urinary excretion.

**Figure S2. Flowchart of participant selection for the internal hospital cohort.**

**Figure S3. Flowchart of participant selection for the NHANES cohort.**

**Table S1. Population characteristics by categories of NFS and FIB-4.**

| **Variables** | NFS ≤0.676 (n = 3297) | NFS >0.676 (n = 1319) | P | FIB-4 ≤3.25 (n = 4462) | FIB-4 >3.25 (n = 154) | P |
| --- | --- | --- | --- | --- | --- | --- |
| **Sex, n (%)** |  |  | *0.982* |  |  | *0.069* |
| Male | 1695 (51.4) | 688 (51.5) |  | 2289 (51.1) | 94 (62.8) |  |
| Female | 1602 (48.6) | 631 (48.5) |  | 2173 (48.9) | 60 (37.2) |  |
| **Age (years), mean ± SE** | 54.9 ± 0.3 | 65.5 ± 0.4 | *< 0.0001* | 57.4 ± 0.3 | 68.4 ± 1.0 | *<0.0001* |
| **Smoke, n (%)** |  |  | *< 0.0001* |  |  | *0.172* |
| Former | 975 (30.6) | 478 (39) |  | 1391 (32.5) | 62 (42.8) |  |
| No | 1709 (51.1) | 678 (49.3) |  | 2321 (51.0) | 66 (40.1) |  |
| Now | 613 (18.3) | 163 (11.7) |  | 750 (16.5) | 26 (17.1) |  |
| **Alcohol, n (%)** |  |  | *< 0.0001* |  |  | *0.358* |
| No | 1406 (36.5) | 723 (48.3) |  | 2059 (39.9) | 70 (34.9) |  |
| Yes | 1891 (63.5) | 596 (51.7) |  | 2403 (60.1) | 84 (65.1) |  |
| **Marital status, n (%)** |  |  | *< 0.0001* |  |  | *0.098* |
| Married | 1856 (59.5) | 697 (57.6) |  | 2476 (59.3) | 77 (52.1) |  |
| Living with partner | 174 (5.9) | 45 (4.1) |  | 211 (5.3) | 8 (8.0) |  |
| Divorced | 441 (13.3) | 162 (11.4) |  | 590 (13.1) | 13 (5.0) |  |
| Never married | 365 (11.0) | 102 (7.1) |  | 455 (9.8) | 12 (13.3) |  |
| Separated | 125 (2.6) | 51 (2.8) |  | 170 (2.7) | 6 (3.0) |  |
| Widowed | 333 (7.7) | 262 (17) |  | 558 (9.9) | 37 (18.6) |  |
| **BMI (kg/m²)** | 31.7 ± 0.2 | 37.4 ± 0.3 | *< 0.0001* | 33.3 ± 0.2 | 30.4 ± 0.7 | *< 0.0001* |
| **Hyperlipidemia, n (%)** |  |  | *0.105* |  |  | *0.253* |
| No | 426 (12.4) | 165 (10.2) |  | 564 (11.6) | 27 (15.9) |  |
| Yes | 2871 (87.6) | 1154 (89.8) |  | 3898 (88.4) | 127 (84.1) |  |
| **HbA1c (%)** | 7.2 ± 0.0 | 7.1 ± 0.1 | *0.138* | 7.2 ± 0.0 | 6.7 ± 0.2 | *0.011* |
| **25 (OH)D (nmol/L)** | 64.1 (46.9, 82.9) | 66 (44.5, 86.4) | *0.7058* | 64.7 (46.5, 83.6) | 68.3 (40.2, 99.5) | *0.4365* |
| **25 (OH)D (ng/mL)** | 25.6 (18.8, 33.2) | 26.4 (17.8, 34.6) |  | 25.9 (18.6, 33.4) | 27.32 (16.1, 39.8) |  |
| **25 (OH)D Tertiles, n (%)** |  |  |  |  |  |  |
| Tertile 1 | 1095 (27.8) | 430 (29.0) | *0.342* | 1482 (28.0) | 43 (30.0) | *0.305* |
| Tertile 2 | 1121 (33.0) | 404 (29.4) |  | 1475 (32.3) | 50 (24.3) |  |
| Tertile 3 | 1081 (39.3) | 485 (41.6) |  | 1505 (39.7) | 61 (45.8) |  |
| **25 (OH)D2 (nmol/L)** | 1.4 (1.4, 1.4) | 1.4 (1.4, 2.2) | *0.0039* | 1.4 (1.4, 1.4) | 1.4 (1.4, 3) | *0.1115* |
| **25 (OH)D2 (ng/mL)** | 0.6 (0.6, 0.6) | 0.6 (0.6, 0.9) |  | 0.6 (0.6, 0.6) | 0.6 (0.6, 1.2) |  |
| **25 (OH)D3 (nmol/L)** | 59.8 (42.5, 78.8) | 58.4 (36.4, 79) | *0.0855* | 59.6 (41.3, 78.4) | 58.8 (35.8, 91.8) | *0.7913* |
| **25 (OH)D3 (ng/mL)** | 24 (17, 31.6) | 23.4 (14.6, 31.7) |  | 23.9 (16.6, 31.4) | 23.6 (13.3, 36.8) |  |
| **25 (OH)D3 Tertiles, n (%)** |  |  |  |  |  |  |
| Tertile 1 | 1054 (25.7) | 469 (32.8) | *0.01* | 1469 (27.4) | 54 (35.4) | *0.059* |
| Tertile 2 | 1123 (33.2) | 402 (28.4) |  | 1481 (32.3) | 44 (20.8) |  |
| Tertile 3 | 1120 (41.1) | 448 (38.7) |  | 1512 (40.3) | 56 (43.8) |  |

Abbreviations: BMI, body mass index; FIB-4, Fibrosis-4 index; NFS, NAFLD Fibrosis Score; HbA1c, hemoglobin A1c; 25(OH)D, serum 25-hydroxyvitamin D; 25(OH)D2, serum 25-hydroxyvitamin D2; 25(OH)D3, serum 25-hydroxyvitamin D3.

**Table S2. Hazard ratios (HRs) and 95% confidence intervals (CIs) for mortality according to NFS and FIB-4 categories.**

|  | HR (95% CI) P value |  |  |
| --- | --- | --- | --- |
| **Variables** | All-cause mortality | Cardiovascular mortality | Diabetes-related mortality |
| NFS | 2.509(1.940, 3.245) <0.0001 | 2.893(2.046, 4.091) <0.0001 | 2.712(1.274, 5.774) 0.010 |
| FIB-4 | 3.131(2.146, 4.569) <0.0001 | 3.441(1.842, 6.427) <0.001 | 4.042(1.449, 11.280) 0.008 |

Abbreviations: HR, hazard ratio; CI, confidence interval. NFS models for all three mortality outcomes were adjusted for sex, smoking status, alcohol consumption, marital status, race/ethnicity, education level, poverty-income ratio, hyperlipidemia, and HbA1c. FIB-4 models for all-cause and cardiovascular mortality were adjusted for the same covariates plus BMI. Because diabetes-related deaths were sparse in the FIB-4 >3.25 stratum, the FIB-4–diabetes-related mortality estimate used a reduced exploratory covariate specification and should not be interpreted as a reliable effect-size estimate.Diabetes-related mortality estimates for FIB-4 should be interpreted cautiously because of the relatively limited number of diabetes-related deaths.

**Table S3. Exploratory hazard ratios (HRs) and 95% confidence intervals (CIs) for mortality according to NFS and FIB-4 categories, stratified by vitamin D tertiles.**

| **Variables** |  | HR (95% CI) P value |  |  |
| --- | --- | --- | --- | --- |
| **NFS** |  | All-cause mortality | Cardiovascular mortality | Diabetes-related mortality |
|  | **25(OH)D** |  |  |  |
|  | Tertile 1 | 3.106(2.092, 4.610) <0.0001 | 3.106(1.785, 5.402) <0.0001 | 3.751(0.955, 14.724) 0.058 |
|  | Tertile 2 | 2.181(1.493, 3.187) <0.0001 | 3.375(1.825, 6.243) <0.001 | 1.774(0.479, 6.564) 0.390 |
|  | Tertile 3 | 2.409(1.689, 3.437) <0.0001 | 2.643(1.441, 4.847) 0.002 | 1.898(0.747, 4.819) 0.178 |
|  | **25(OH)D3** |  |  |  |
|  | Tertile 1 | 2.666(1.739, 4.086) <0.0001 | 2.614(1.556, 4.393) <0.001 | 2.848(0.865, 9.373) 0.085 |
|  | Tertile 2 | 2.583(1.704, 3.917) <0.0001 | 2.839(1.584, 5.091) <0.001 | 2.077(0.587, 7.351) 0.257 |
|  | Tertile 3 | 2.402(1.651, 3.494) <0.0001 | 3.355(1.921, 5.859) <0.0001 | 2.008(0.710, 5.682) 0.189 |
| **FIB-4** |  |  |  |  |
|  | **25(OH)D** |  |  |  |
|  | Tertile 1 | 3.753(1.847, 7.626) <0.001 | 5.600(1.757, 17.851) 0.004 | 10.531(2.398, 46.244) 0.002 |
|  | Tertile 2 | 2.290(1.213, 4.323) 0.011 | 1.926(0.608, 6.097) 0.265 | 1.005(0.113, 8.932) 0.997 |
|  | Tertile 3 | 3.054(1.551, 6.013) 0.001 | 2.396(0.714, 8.035) 0.157 | 4.333(0.792, 23.698) 0.091 |
|  | **25(OH)D3** |  |  |  |
|  | Tertile 1 | 3.612(1.954, 6.678) <0.0001 | 5.944(2.224, 15.883) <0.001 | 9.766(3.621, 26.340) <0.0001 |
|  | Tertile 2 | 2.543(1.539, 4.200) <0.001 | 1.811(0.670, 4.894) 0.242 | 1.197(0.118, 12.196) 0.879 |
|  | Tertile 3 | 2.865(1.392, 5.898) 0.004 | 2.298(0.691, 7.643) 0.175 | 2.873(0.235, 35.135) 0.409 |

Abbreviations: HR, hazard ratio; CI, confidence interval; 25(OH)D, serum 25-hydroxyvitamin D; 25(OH)D2, serum 25-hydroxyvitamin D2; 25(OH)D3, serum 25-hydroxyvitamin D3; FIB-4, Fibrosis-4 index; NFS, NAFLD Fibrosis Score.

NFS models for all three mortality outcomes were adjusted for sex, smoking status, alcohol consumption, marital status, race/ethnicity, education level, poverty-income ratio, hyperlipidemia, and HbA1c. FIB-4 models for all-cause and cardiovascular mortality were adjusted for the same covariates plus BMI. Diabetes-related mortality estimates in the FIB-4 >3.25 subgroup, especially after stratification by 25(OH)D3 tertiles, should be interpreted as exploratory sparse-event estimates because only 5 diabetes-related deaths were observed in this subgroup, with 3, 1, and 1 events across increasing 25(OH)D3 tertiles.

**Table S4. Schoenfeld residual tests for the proportional hazards assumption in the main Cox regression models.**

| Model | Main exposure | Main exposure P value | Global P value |
| --- | --- | --- | --- |
| **NFS - all-cause mortality** | NFS | 0.35 | 0.38 |
| **NFS - cardiovascular mortality** | NFS | 0.42 | 0.77 |
| **NFS- diabetes-related mortality** | NFS | 0.15 | 0.44 |
| **FIB-4 - all-cause mortality** | FIB-4 | 0.81 | 0.31 |
| **FIB-4 - cardiovascular mortality** | FIB-4 | 0.74 | 0.61 |
| **FIB-4- diabetes-related mortalitya** | FIB-4 | 0.85 | 0.46 |

Abbreviations: NFS, NAFLD Fibrosis Score; FIB-4, Fibrosis-4 index. The proportional hazards assumption was assessed using Schoenfeld residuals. Models were evaluated using the corresponding covariate-adjusted specifications used in the primary Cox analyses. For the FIB-4–diabetes-related mortality model, a reduced diagnostic specification was used only for Schoenfeld-residual assessment because the fully adjusted specification was numerically unstable for proportional-hazards diagnostics in the setting of sparse events. This diagnostic specification did not replace the HR estimates reported in Tables S2 and S3.The proportional hazards assumption was nonetheless satisfied for the principal exposure term (P = 0.85) and in the global test (P = 0.46).

**Table S5. Multiple-imputation sensitivity analysis for the associations of vitamin D metabolites with NFS and FIB-4 in NHANES.**

|  | **NFS** |  | **FIB-4** |  |
| --- | --- | --- | --- | --- |
| **Variables** | **OR (95% CI)** | P value | OR (95% CI) | P value |
| **25(OH)D (continuous)** | 0.998(0.994, 1.002) | 0.264 | 1.000(0.991, 1.008) | 0.911 |
| **25(OH)D** **Tertiles** |  |  |  |  |
| Tertile 1 | Reference |  | Reference |  |
| Tertile 2 | 0.733(0.545, 0.986) | 0.043 | 0.472(0.239, 0.932) | 0.034 |
| Tertile 3 | 0.829(0.605, 1.138) | 0.251 | 0.661(0.373, 1.169) | 0.159 |
| **25(OH)D2 (continuous)** | 1.009(1.003, 1.014) | 0.004 | 1.008(0.999, 1.016) | 0.080 |
| **25(OH)D2 Tertiles** |  |  |  |  |
| Tertile 1 | Reference |  | Reference |  |
| Tertile 2 | 1.104(0.883, 1.381) | 0.389 | 0.702(0.408, 1.207) | 0.205 |
| Tertile 3 | 1.405(1.133, 1.743) | 0.003 | 1.191(0.673, 2.107) | 0.551 |
| **25(OH)D3 (continuous)** | 0.995(0.991, 0.999) | 0.026 | 0.997(0.988, 1.006) | 0.493 |
| **25(OH)D3 Tertiles** |  |  |  |  |
| Tertile 1 | Reference |  | Reference |  |
| Tertile 2 | 0.639(0.473, 0.864) | 0.005 | 0.356(0.188, 0.676) | 0.002 |
| Tertile 3 | 0.654(0.475, 0.901) | 0.011 | 0.499(0.291, 0.855) | 0.014 |

Abbreviations: OR, odds ratio; CI, confidence interval; 25(OH)D, serum 25-hydroxyvitamin D; 25(OH)D2, serum 25-hydroxyvitamin D2; 25(OH)D3, serum 25-hydroxyvitamin D3; FIB-4, Fibrosis-4 index; NFS, NAFLD Fibrosis Score.

NFS model was adjusted for sex, smoking status, alcohol consumption, marital status, HbA1c, race/ethnicity, education level, poverty-income ratio, and hyperlipidemia. FIB-4 model was adjusted for the same covariates plus BMI. Missing covariates included marital status, education level, and poverty-income ratio; these variables were imputed using multiple imputation by chained equations with 20 imputed datasets. Outcome variables, exposure variables, and covariates without missingness were not imputed.

**Table S6. Subgroup sensitivity analyses of the association between serum 25(OH)D3 and advanced fibrosis in the NHANES cohort.**

| **Subgroup** | **Outcome** | **OR (95% CI)** | **P value** |
| --- | --- | --- | --- |
| **Age ≥ 60 years** | | | |
|  | NFS > 0.676 | 0.994 (0.990–0.998) | 0.008 |
|  | FIB-4 > 3.25 | 0.995 (0.987–1.003) | 0.255 |
| **BMI < 30 kg/m²** | | | |
|  | NFS > 0.676 | 0.999 (0.992–1.007) | 0.865 |
|  | FIB-4 > 3.25 | 1.000 (0.987–1.013) | 0.977 |
| **BMI ≥ 30 kg/m²** | | | |
|  | NFS > 0.676 | 0.996 (0.991–1.002) | 0.177 |
|  | FIB-4 > 3.25 | 0.994 (0.978–1.011) | 0.494 |

Odds ratios are expressed per 1 ng/mL increase in serum 25(OH)D3. Models were adjusted using the same covariate sets as the primary NHANES logistic regression analyses. The FIB-4 models were additionally adjusted for BMI, except in BMI-stratified analyses where BMI was used as the stratification variable. NFS, NAFLD Fibrosis Score; FIB-4, Fibrosis-4 index; BMI, body mass index; OR, odds ratio; CI, confidence interval.

**Table S7. Sensitivity analyses excluding participants with the highest fibrosis-score values in the NHANES cohort.**

| **Sensitivity analysis** | **Exposure** | **OR (95% CI)** | **P value** |
| --- | --- | --- | --- |
| Excluding highest decile of NFS | 25(OH)D3 (continuous) | 0.995 (0.990–1.000) | 0.008 |
| Excluding highest decile of FIB-4 | 25(OH)D3 (continuous) | 1.000 (0.998–1.002) | 0.912 |

Odds ratios are expressed per 1 ng/mL increase in serum 25(OH)D3. In each analysis, participants in the highest decile of the corresponding fibrosis score were excluded before refitting the primary NHANES logistic regression model. NFS, NAFLD Fibrosis Score; FIB-4, Fibrosis-4 index; OR, odds ratio; CI, confidence interval.

**Table S8. Numbers of deaths among participants with advanced fibrosis according to 25(OH)D3 tertiles.**

| **Fibrosis category** | **25(OH)D3 tertile** | **No. participants, n** | **All-cause deaths, n** | **Cardiovascular deaths, n** | **Diabetes-related deaths, n** |
| --- | --- | --- | --- | --- | --- |
| **NFS > 0.676** | Overall | 1319 | 344 | 116 | 27 |
|  | Tertile 1 | 469 | 131 | 43 | 13 |
|  | Tertile 2 | 402 | 110 | 37 | 7 |
|  | Tertile 3 | 448 | 103 | 36 | 7 |
| **FIB-4 > 3.25** | Overall | 154 | 65 | 19 | 5 |
|  | Tertile 1 | 54 | 24 | 9 | 3 |
|  | Tertile 2 | 44 | 21 | 5 | 1 |
|  | Tertile 3 | 56 | 20 | 5 | 1 |

Data are shown as the number of participants and the number of deaths during follow-up among participants in the high-risk fibrosis categories, overall and across tertiles of serum 25(OH)D3. Diabetes-related mortality estimates in the FIB-4 > 3.25 subgroup should be interpreted cautiously because only 5 diabetes-related deaths were observed, with 3, 1, and 1 events across increasing 25(OH)D3 tertiles. NFS, NAFLD Fibrosis Score; FIB-4, Fibrosis-4 index.

**Table S9. Comparison of covariate adjustment between the internal hospital cohort and NHANES.**

| Covariate | Internal hospital cohort | NHANES cohort | Comment |
| --- | --- | --- | --- |
| Age | Included in NFS/FIB-4 calculation | Included in NFS/FIB-4 calculation | Not additionally adjusted when part of the score |
| Sex | Adjusted | Adjusted | Available in both cohorts |
| BMI | Adjusted in FIB-4 models; excluded from NFS models | Adjusted in FIB-4 models; excluded from NFS models | Excluded from NFS models because BMI is a component of NFS |
| Smoking status | Adjusted | Adjusted | Available in both cohorts |
| Alcohol consumption | Adjusted | Adjusted | Available in both cohorts |
| Marital status | Adjusted | Adjusted | Available in both cohorts |
| Season of blood draw | Adjusted | Not adjusted | Available only in the internal hospital cohort |
| Race/ethnicity | Not available | Adjusted | NHANES-specific covariate |
| Education level | Not available | Adjusted | NHANES-specific covariate |
| Poverty-income ratio | Not available | Adjusted | NHANES-specific socioeconomic covariate |
| HbA1c | Adjusted | Adjusted | Available in both cohorts |
| Fatty liver | Adjusted | Not adjusted | Available in the internal hospital cohort |
| Coronary heart disease | Adjusted | Not included in final NHANES model | Cohort-specific availability/definition |
| Hypertension | Adjusted | Not included in final NHANES model | Cohort-specific adjustment |
| Triglycerides | Adjusted | Not included in final NHANES model | Cohort-specific adjustment |
| LDL-C | Adjusted | Not included in final NHANES model | Cohort-specific adjustment |
| Hyperlipidemia | Not included as a separate final covariate | Adjusted | NHANES final model used hyperlipidemia status |

Covariate adjustment differed between the two cohorts because of differences in data availability and cohort design. The internal hospital cohort included clinically available variables such as season of blood draw, fatty liver, CHD, hypertension, triglycerides, and LDL-C, whereas the NHANES models included survey-available sociodemographic variables such as race/ethnicity, education level, and poverty-income ratio. This table is intended to transparently summarize covariate availability and adjustment rather than to imply identical adjustment across cohorts.

**Table S10. Comparison of NHANES participants included in complete-case covariate-adjusted analyses and those excluded because of missing model covariates.**

| **Variable** | **Included (n = 4,161)** | **Excluded (n = 455)** | **P value** |
| --- | --- | --- | --- |
| Age (years) | 59.67 ± 13.35 | 62.33 ± 13.04 | <0.001 |
| BMI (kg/m2) | 32.58 ± 7.71 | 31.89 ± 7.03 | 0.084 |
| HbA1c (%) | 7.26 ± 1.75 | 7.28 ± 1.69 | 0.200 |
| 25(OH)D3 (nmol/L) | 58.4 ± 28.73 | 57.8 ± 28.44 | 0.683 |
| Male sex, n (%) | 2165 (52.0) | 218 (47.9) | 0.105 |
| NFS >0.676, n (%) | 1169 (28.1) | 150 (33.0) | 0.033 |
| FIB-4 >3.25, n (%) | 142 (3.4) | 12 (2.6) | 0.461 |

Values are mean ± SD or n (%). P values were calculated using t-tests for continuous variables and chi-square tests for categorical variables. BMI, body mass index; HbA1c, hemoglobin A1c; NFS, NAFLD Fibrosis Score; FIB-4, Fibrosis-4 index.
